# The Role of 17β-Estradiol and Estrogen Receptors in Regulation of Ca^2+^ Channels and Mitochondrial Function in Cardiomyocytes

**DOI:** 10.3389/fendo.2019.00310

**Published:** 2019-05-15

**Authors:** Shokoufeh Mahmoodzadeh, Elke Dworatzek

**Affiliations:** ^1^Department of Molecular Muscle Physiology, Max-Delbrueck-Center for Molecular Medicine in the Helmholtz Association, Berlin, Germany; ^2^DZHK (German Centre for Cardiovascular Research), Partner Site Berlin, Berlin, Germany; ^3^Institute of Gender in Medicine, Charité Universitaetsmedizin, Berlin, Germany

**Keywords:** estrogen, estrogen receptor, G-protein-coupled estrogen receptor, cardiomyocytes, sex difference, cardiac mitochondrial function, cardiac Ca^2+^ ion channel

## Abstract

Numerous epidemiological, clinical, and animal studies showed that cardiac function and manifestation of cardiovascular diseases (CVDs) are different between males and females. The underlying reasons for these sex differences are definitely multifactorial, but major evidence points to a causal role of the sex steroid hormone 17β-estradiol (E2) and its receptors (ER) in the physiology and pathophysiology of the heart. Interestingly, it has been shown that cardiac calcium (Ca^2+^) ion channels and mitochondrial function are regulated in a sex-specific manner. Accurate mitochondrial function and Ca^2+^ signaling are of utmost importance for adequate heart function and crucial to maintaining the cardiovascular health. Due to the highly sensitive nature of these processes in the heart, this review article highlights the current knowledge regarding sex dimorphisms in the heart implicating the importance of E2 and ERs in the regulation of cardiac mitochondrial function and Ca^2+^ ion channels, thus the contractility. In particular, we provide an overview of *in-vitro* and *in-vivo* studies using either E2 deficiency; ER deficiency or selective ER activation, which suggest that E2 and ERs are strongly involved in these processes. In this context, this review also discusses the divergent E2-responses resulting from the activation of different ER subtypes in these processes. Detailed understanding of the E2 and ER-mediated molecular and cellular mechanisms in the heart under physiological and pathological conditions may help to design more specifically targeted drugs for the management of CVDs in men and women.

## Introduction

Cardiovascular Diseases (CVDs) are one of the top age-associated chronic diseases with growing importance due to the dramatic increase in life expectancy (1) and are the leading cause of mortality in men and women worldwide (2). In the vast majority of CVDs, there are well described sex differences in the incidence, pathophysiology, and outcomes of diseases (3). As result of these observations, research over the last few decades has focused on the contribution of sex steroid hormones, specifically 17β-estradiol (E2), on the cardiovascular system and mechanistic pathways in the diseased heart.

Calcium (Ca^2+^) is a key player in the regulation of myocardial contraction and the deregulation of Ca^2+^ signaling due to the alteration of Ca^2+^ ion channels function in cardiomyocytes is highly associated with the development of cardiac diseases, such as heart failure (4). Just like Ca^2+^, mitochondria play an essential role in the regulation of energy metabolism of the heart, and defects of mitochondrial function also lead to the development and progression of cardiovascular diseases (5, 6). This review article provides an overview of the current knowledge regarding the sex differences in cardiac health and disease with the focus on the sexually dimorphic effects of E2 and estrogen receptors (ERs) in the regulation of cardiomyocyte's Ca^2+^ ion channels and mitochondrial function.

## The Role of 17β-Estradiol in the Heart

Epidemiological data suggest that premenopausal women are protected from the incidence of CVDs as well as from resulting morbidity and mortality compared with age-matched men, but that this protection is lost after menopause (7–9). This led to the generally accepted conclusion that the sex hormone E2 protects against CVDs in women (10). However, recent large-scale clinical trials revealed conflicting data about the effect of E2 on CVDs, which is still a matter of intense debate. For example, several observational studies such as the *Nurse's Health Study* showed that postmenopausal women with hormone replacement therapy (HRT) have a lower rate of CVDs and cardiac death, compared to women without HRT (11–14). In contrast, the *Women Health Initiative* (WHI) and the *Heart and Estrogen/Progestin Replacement Study* (HERS I and II) showed that HRT has no obvious beneficial effect on CVDs, and may actually increase the risk and events of CVDs in postmenopausal women (15–19). The reasons for this paradox remain unclear and many potential factors, such as the study design and subject characteristics, the form of applied E2 (which type of E2, combination of E2 with progestin), the dosage and pharmacokinetics of the HRT used, and the statistical power to address cardiac risk factors may contribute to the discrepant results and to the adverse outcome of HRT (20–22). In addition, another reason for the contradictory data could be the timing of HRT initiation. Recent studies such as the *Kronos Early Estrogen Prevention Study* (KEEPS) and the *Early vs. Late Intervention Trial with Estradiol* (ELITE) addressed the question of the so-called “timing hypothesis.” They showed significant beneficial cardiovascular effects in women who initiated HRT in the early postmenopause vs. late menopause period (19, 23, 24), indicating the importance of the time point of HRT-application.

Modulatory effects of E2 on CVDs in men have also been reported (25, 26). In men with E2 deficiency due to a mutation in the cytochrome P450 aromatase gene (*Cyp19a1*), which catalyzes the aromatization of androgens to E2, or E2 resistance, caused by a point mutation in the ERα gene (*ESR1)*, the following have been reported: increased total cholesterol level, the development of insulin resistance, impaired glucose tolerance, type 2 diabetes mellitus, and impaired vasodilatation (27–31). These data suggest that the physiological concentrations of E2 might reduce the risk of CVDs in men. Indeed, men with abnormally low (≤13 pg/mL) and abnormally high (≥37 pg/mL) E2-levels have been found to show the highest death rates from congestive heart failure (32). By contrast, individuals with levels of E2 in the range of 22–30 pg/mL had the least number of deaths over a 3-year period. However, the precise role of E2 in men in CVDs remains questionable (33).

## Actions of 17β-Estradiol and Estrogen Receptors

E2 belongs together with Estrone (E1) and Estriol (E3) to the group of sex steroids called Estrogens. Thereby, E2 is the predominant and most biologically active form (34). Estrogens have traditionally been associated with the female reproductive development and function, but it is now well-established that they also regulate male reproductive organs and play a physiological role in multiple organs in both sexes (26). In healthy premenopausal women, ovaries are the primary site of E2 production, and in men, E2 is produced in small amounts by the testes. E2 is also synthesized in a number of extragonadal tissues, through the conversion of testosterone by cytochrome p450 aromatase in both sexes, including bone, breast, adipose tissue, and the brain (35). There is increasing evidence that the aromatase is also expressed in the heart tissue and that E2 can also be produced locally in cardiac cells (36–39), suggesting that local cardiac E2 synthesis by aromatase plays a role in the E2-mediated effects on CVDs.

**Figure 1:**
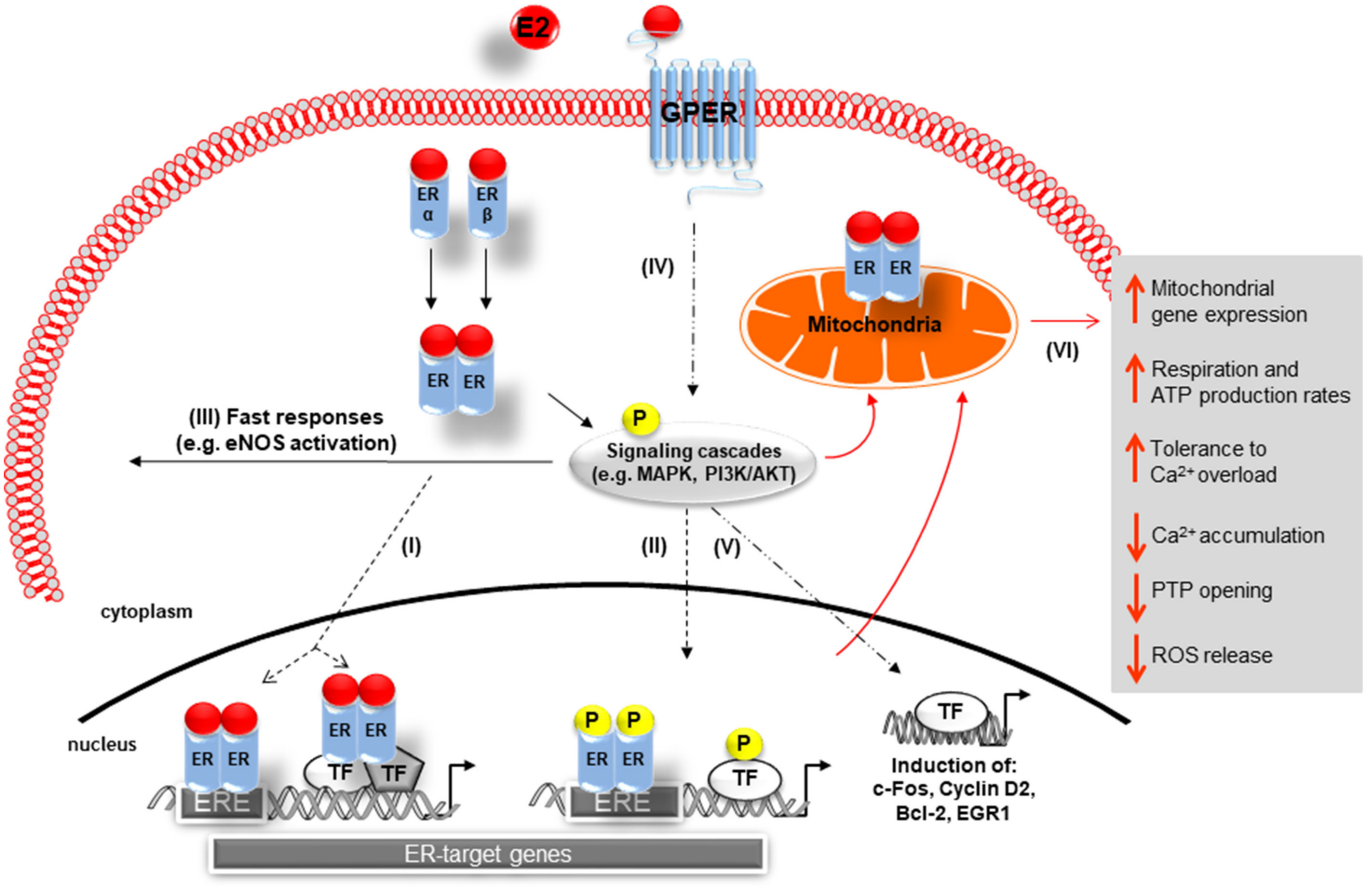
Schematic representation of 17β-Estradiol induced estrogen receptor-alpha, -beta, and G-protein-coupled estrogen receptor signaling. Genomic pathway: **(I)** The E2/ER complex can bind to estrogen response elements (ERE) within the promoter of target genes or regulates gene transcription by interacting with other transcription factors (TF), e.g., AP-1 and Sp1. **(II)** In addition, E2/ER activate signaling transduction pathways, leading to phosphorylation of ER or other bound transcription factors modulating gene expression. In the non-genomic action: **(III)** E2-activated ER lead to rapid tissue responses via phosphorylation of cytosolic signaling cascades. **(IV)** GPER predominantly mediates rapid, non-genomic E2 signaling by the involvement of several kinases, ion channels, and second messengers. **(V)** GPER is also involved in gene expression regulation. **(VI)** E2 initiated cellular and mitochondrial ER/GPER genomic and non-genomic actions modulate mitochondrial respiration, ATP production, and ROS formation (indicated by red arrows). E2, 17β-estradiol; ER, estrogen receptor alpha and beta ERE, estrogen response element; TF, transcription factor; P, phosphorylation; GPER, G-protein-coupled estrogen receptor; Ca^2+^, calcium, PTP, permeability transition pore; MAPK, mitogen-activated protein kinases; PI3K/AKT, phosphoinositide 3-kinase-serin/threonine-specific kinase B; eNOS: endothelial nitric oxide synthase.

The physiological effects of E2 are predominantly mediated via estrogen receptor alpha (ERα) and beta (ERβ), which are members of the nuclear receptor superfamily (Figure 1) (40). Both receptors carry similar structural domains, however, they differ in their DNA- and ligand-binding regions, which are of crucial importance for their diverse transcriptional actions (41). E2-activated ERs can act as ligand-induced transcription factors inducing changes in transcription of E2 target genes, a process referred to as genomic actions. Here the binding of E2 to the ERs results in homo- or heterodimerization of ER and their translocation into the nucleus of cells. The E2/ER complex either binds to estrogen response elements (ERE) within the promoter of target genes or regulates gene transcription by interacting with other transcription factors, e.g., AP-1 and Sp1 (Figure 1I) (34, 42–44). Additionally, E2-bound ERs can also activate multiple signal transduction pathways, e.g., *mitogen-activated protein kinases ERK1/2* and -*p38* (ERK1/2-MAPK, p38-MAPK) as well as *phosphoinositide 3-kinase-serin/threonine-specific kinase* B (PI3K/AKT), which in turn phosphorylate ERs (45–47) or other promoter bound transcription factors that are involved in the regulation of E2-target gene expression (Figure 1II) (48–51). Moreover, through non-genomic actions, E2 rapidly mediates its effects by activation of ERs located in or adjacent to the plasma membrane, which in turn can activate different signal transduction cascades, such as PI3K/AKT and MAPK, leading for example to cytosolic eNOS activation (Figure 1III) (52, 53).

## Estrogen Receptors in the Heart

Both ERs are localized in different cardiac cells such as cardiomyocytes, endothelial cells, smooth muscle cells, and cardiac fibroblasts in human hearts from both sexes (54, 55). Studies in rodents also showed that both ER are expressed in whole heart tissue from males and females (36, 39, 56–58). Recent observations from Pugach et al. showed that only ERα, but not ERβ, is expressed in left ventricular heart tissue from mice and isolated rat cardiomyocytes (59). However, there are several other studies that not only showed the expression of both ERs in cardiomyocytes of rodents but also their functional activity on genomic and non-genomic levels (36, 60–67).

Recent reports showed that E2 can signal through a third protein, the G-protein-coupled estrogen receptor (GPER), formerly known as GPR30, a membrane receptor with seven transmembrane spanning domains (68, 69). GPER is strongly expressed in both male and female human and rat cardiac tissue (70–73). Specifically, GPER is present in smooth muscle cells (74, 75), endothelial cells (76), cardiac fibroblasts (77), and cardiomyocytes (70). GPER has been implicated predominantly in the rapid, non-genomic E2 signaling by the involvement of several kinases, ion channels and second messengers in a wide variety of cell types (Figure 1IV) (69, 78–80). However, effects on gene expression, i.e., induction of c-fos, cyclin D2, Egr-1, and Bcl-2 expression, have also been described (81–85).

## Association of Genetic Alterations and Polymorphisms of the Estrogen Receptor Genes and Cardiovascular Disease

Studies showed that mutations in the genes coding for ERα and ERβ are associated with differences in heart morphology, such as increased left ventricular mass and wall thickness (86, 87). Furthermore, single nucleotide polymorphisms (SNPs) in both ERα and ERβ have been shown to be associated with the susceptibility for CVDs. Most of the studies analyzing ERα focused on two SNPs: c.454-397T>C (rs2234693) and c.454-351A>G (rs9340799) located in the first intron of the ERα gene and 46 bp apart from each other (88). In fact, the ERα variant rs2234693 was linked to coronary heart disease among Finnish men (89), whereas a study of a Dutch cohort showed that ERα variants, rs2234693, and rs9340799, were associated with increased risk of myocardial infarction (MI) and ischemic heart disease (IHD) only in postmenopausal women, but not in men (90). In contrast, in a prospective study in men and women from the population based offspring cohort of the Framingham Heart Study showed that individuals of both sexes carrying the rs2234693 genotype have substantial increase in risk of MI (91). The authors confirmed their findings in men in a latter study, including 7,000 men in five cohorts from four countries (92). In contrast, other studies found no association between these two SNPs or their haplotypes and MI or risk of CVD in either women or men (88, 93–95). Additionally, the absence of ERα in human vascular smooth muscle cells in premenopausal women (96) or the reduced ERα expression, due to methylation of the receptor with increasing age, is associated with the development of atherosclerosis in the cardiovascular system (97).

For ERβ, the SNP variant rs1271572 was associated with increased risk of MI in Spanish men (98), while Rexrode et al. identified this ERβ variant to be associated with increased risk of MI in women only (99). Additionally, this study showed the linkage of another ERβ variant, the rs1256049, with reduced risk of CVDs or MI in women (99).

The reasons for the inconsistency in data regarding the SNPs within the genes of ERα and ERβ could be due to the limited power within the studies, differences in methodology and study population (93). Despite the inconsistent findings, together these studies provide support for a relationship between ERα and ERβ polymorphisms and the risk of CVDs in men and women. The underlying mechanisms responsible for the phenotype associated with these genetic variants are not yet known. It is recognized that ER-SNPs can cause changes in E2-mediated downstream gene expression and signaling, which can alter the effects of E2 on the heart (100) and may be one possible explanation for the observed effects on the cardiovascular system. In contrast to ERα and ERβ, there are no studies so far regarding the association of polymorphisms within the GPER gene and cardiac risk in humans.

## The Role of Estrogen Receptors in Animal Models for Human Cardiovascular Diseases

The physiology of E2-actions through its multiple receptors is diverse and highly complex. The detailed understanding of their effects and underlying molecular mechanisms are essential for future therapeutic applications in humans. In order to clarify remaining questions regarding the functions of each individual receptor within the heart, different mouse models with a deficiency or overexpression of ERα, ERβ, and GPER have been generated (101, 102).

### ERα

At the basal level, male and female whole body ERα-deficient (ERKO)-mice are obese and insulin resistant (103). They also exhibit altered cardiac substrate preference with a reduction in glucose uptake indicating that ERα is required to maintain glucose utilization in the mouse heart (104). However, ERKO-mice do not show any cardiac dysfunction under physiological conditions. Following cardiac injuries, such as ischemic-reperfusion (I/R) injury or induced chronic MI, male and female ERKO-mice show increased cardiomyocyte cell death, mitochondrial damage, marked coronary edema, decreased coronary flow rate, and poorer functional recovery of contractility (+dP/dt) and compliance (-dP/dt) in comparison to wild type (WT)-mice (105, 106). These data suggest a cardiac protective role of ERα in both sexes after I/R or MI. In contrast, following pressure overload induced myocardial hypertrophy by transverse aortic constriction, female ERKO-mice developed myocardial hypertrophy to an identical degree as that seen in WT females, indicating that ERα is not essential for the attenuation of pressure overload induced hypertrophy observed in females (107, 108).

Analysis of mice hearts carrying a cardiomyocyte-specific deletion of ERα (cs-ERKO) revealed variations in the expression of genes involved in metabolism, cell growth and differentiation, muscle architecture, and relaxation compared to WT-mice (109). Furthermore, under basal conditions hearts from male and female cs-ERKO-mice showed reduction of left ventricular mass accompanied by decreased left ventricle (LV) diameter compared with WT-mice. These data are in line with published findings in mice with cardiomyocyte specific ERα-overexpression (csERα-OE), showing that constitutive ERα-overexpression in cardiomyocytes resulted in higher left ventricular mass and increased ventricular volumes. In addition, greater cardiomyocyte length, augmented expression of hypertrophy-associated genes such as *nppa* and *nppb, but* no fibrosis development was observed (65). In agreement with these data, findings from ovariectomized (OVX) mice also emphasize an E2-dependent role of ERα on regulation of cardiomyocyte size and cardiac growth in healthy mice (110). Overall, these findings indicate that ERα restricted to the cardiomyocytes is associated with the growth in cardiac mass in both sexes.

Interestingly, the use of csERα-OE mice demonstrated that ERα provides cardioprotection in female mice by enhancing neovascularization and impairment of cardiac remodeling in response to cardiac ischemic injury (65). All together, these findings indicate that in the female sex, ERα in cardiomyocytes may have a therapeutic potential in the treatment of ischemic heart disease, leading to more efficient cardiac repair after cardiac injury.

### ERβ

In contrast to ERKO-mice, male and female ERβ-deficient (BERKO)-mice show a mild metabolic phenotype characterized by increased cortical bone formation and loss of trabecular bone (111). In addition, ERβ deficiency protects against diet-induced insulin resistance and glucose intolerance (112). However, with increasing age, BERKO-mice show cardiac hypertrophy, hypertension, and pathology in other cell types as they age (113–115). Additionally, BERKO-mice develop severe cardiomyopathy with a disarray of cardiomyocytes, a disruption of intercalated discs, an increase in number and size of gap junctions, and alteration in nuclear structure (114).

Several studies in BERKO-mice demonstrate the relevant role of ERβ in male and female mice after cardiac injury. The lack of ERβ significantly decreased post-ischemic cardiac recovery and therefore myocardial function in female, but not male, mice (116). In OVX mice subjected to MI, E2-treatment did not reduce infarct size in female BERKO-mice, as observed in ERKO- and WT-mice (117). In line with these data, Pelzer et al. reported that OVX BERKO-mice subjected to chronic MI showed increased mortality rates and aggravated signs of heart failure (118). These observations support the protective role of ERβ in response to I/R or MI in females. Following transverse aortic constriction, increase in left ventricular mass was not attenuated by E2-supplementation in OVX BERKO- as observed in WT- and ERKO-mice (108). Indeed, it has been shown that female BERKO-mice responded to transverse aortic constriction, as well as in the deoxycorticosterone acetate-salt mouse model, with a significantly higher increase in myocardial hypertrophy, marked increase in left ventricular diameters, increased cardiomyocyte size and apoptosis compared with female WT-mice (107, 119, 120). Fliegner et al. showed in male mice lacking ERβ significantly higher cardiomyocyte hypertrophy, increased myocyte apoptosis and faster progression toward heart failure (120). Thus, under pressure overload the loss of ERβ is detrimental for both males and females.

In a mouse model with a cardiomyocyte specific ERβ-overexpression (csERβ-OE), under basal conditions there were no observed differences in heart weight, morphology, and function in males and females (66). Interestingly, the overexpressed ERβ was located within the cytoplasm and nuclei of cardiomyocytes (66), while in csERα-OE mice the ERα protein was mainly located within the nuclei of cardiomyocytes (65). In response to MI, csERβ-OE exhibited improved survival in female and male mice compared to the WT counterparts (66). This was due to attenuated increase in heart weight and LV dilatation as well as improved systolic and diastolic function. In addition, both male and female csERβ-OE mice had a lower reduction of sarcoplasmic/endoplasmic reticulum Ca^2+^-ATPase 2a (SERCA2a) expression, suggesting less reduction in diastolic Ca^2+^-reuptake into the sarcoplasmic reticulum post-MI. Most of these functional parameters were improved in both sexes by csERβ-OE; however, the effect on LV volumes and ejection fraction was more pronounced in males than females. This was possibly due to reduced cardiac remodeling with lower cardiac fibrosis and lower expression of fibrosis markers (collagen I and III, periostin and miR-21), which was observed particularly in male csERβ-OE hearts after MI.

### GPER

There are several studies stating the phenotype of mice lacking GPER (101). The studies of GPER-KO-mice over the last decade revealed that GPER deficient mice show under basal conditions multiple physiological alterations, including obesity (75), insulin resistance, glucose intolerance, and increase in blood pressure (121). Interestingly, it has been reported that male, but not female, GPER-KO-mice show impaired cardiac function with enlarged LV and decreased +dP/dt and –dP/dt (122) or decreased ejection fraction and fractional shortening with increasing age (123). Under cardiac stress, one study reported in a mouse model of I/R that male WT-, ERKO-, and BERKO-mice respond to E2-treatment with an improved recovery and reduced infract size. However, the application of E2 to male GPER-KO-mice did not lead to observed cardioprotection after I/R (80).

A recent study in mice with a cardiomyocyte-specific GPER-KO (csGPER-KO) revealed under basal conditions adverse alterations in cardiac structure and impaired systolic and diastolic function in both sexes, in comparison to WT-mice, with more profound increases in LV dimensions, and wall-thinning among male KO-mice (124). Using DNA microarray analysis, the authors found differential expression profiles of genes affecting multiple transcriptional networks with marked differences in respect to sex and cardiomyocyte-specific GPER deletion. In detail, mitochondrial genes were enriched in cardiomyocytes from female GPER-KO- compared to female WT-mice, but not in male. In contrast, inflammatory response genes were enriched in GPER-KO- vs. WT-cardiomyocytes from male but not female mice (124, 125).

Although studies with transgenic ER mice failed to provide a clear consensus regarding the physiological and pathological roles of ERs, they suggest that each of the ER subtypes play a protective role in the heart.

## The Role of 17β-Estradiol and Estrogen Receptors in Regulation of Ca^2+^ Channels and Contractility in Cardiomyocytes

**Figure 2:**
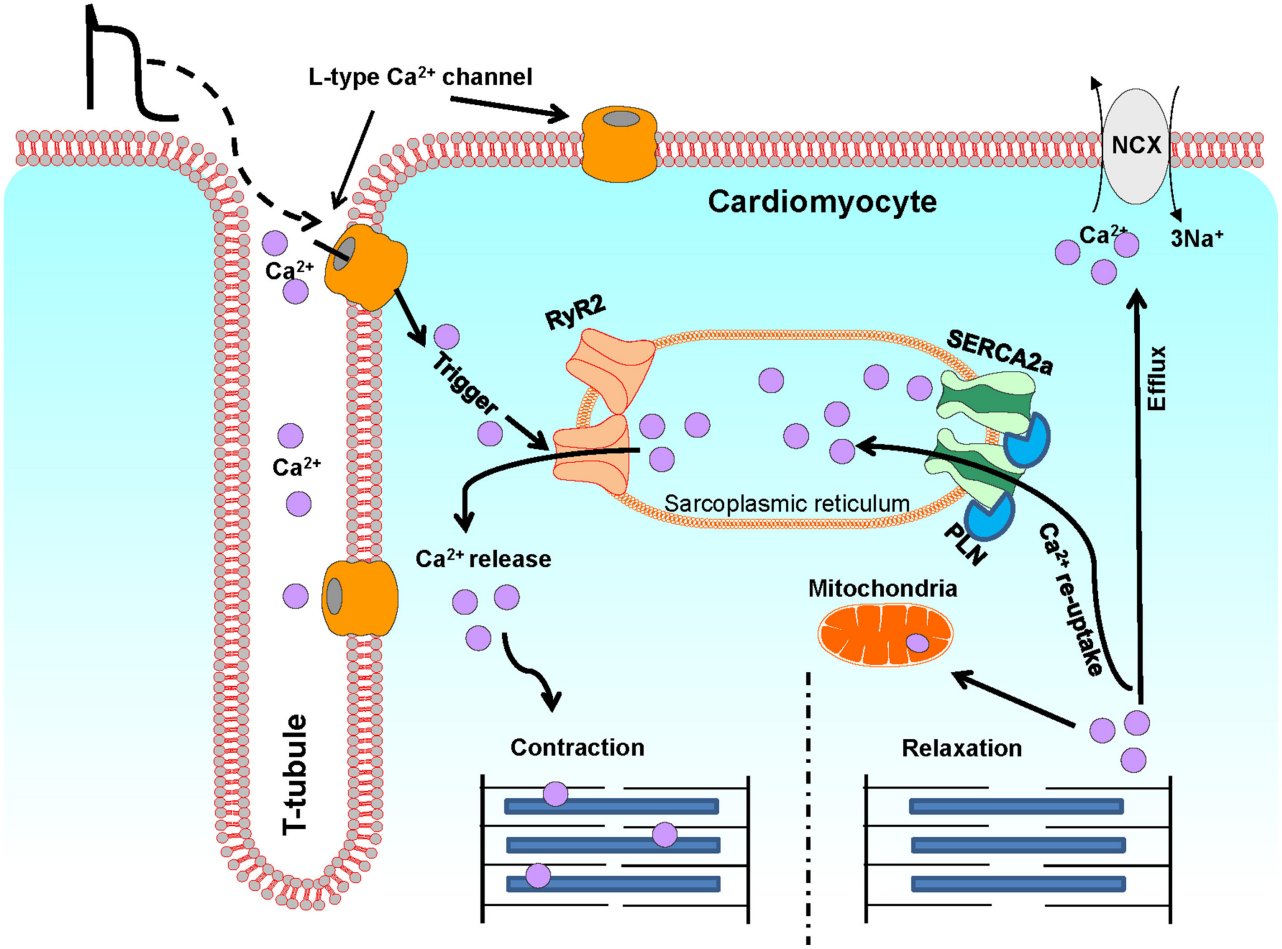
A schematic illustration of Ca^2+^ fluxes during excitation-contraction in ventricular cardiomyocytes. This diagram depicts the most representative protein complexes and intercellular organelles involved in the cardiac excitation-contraction coupling. Ca^2+^, calcium; SR, sarcoplasmic reticulum; M, Mitochondria; LTCC, L-type Ca^2+^ channel; RyR2, Ryanodine receptor 2; SERCA2a, Sarcoplasmic reticulum Ca^2+^ATPase 2a; PLN, Phospholamban; NCX, Na+/Ca^2+^ exchanger.

Ca^2+^ is a critical regulator of myocardial function. Ca^2+^ regulates contraction, and deregulation of Ca^2+^ signaling has been associated with cardiac dysfunction and pathology such as arrhythmias and heart failure (4). In cardiomyocytes, Ca^2+^ levels are tightly regulated via the excitation-contraction (EC) coupling pathway (Figure 2). During action potential, in response to depolarization, Ca^2+^ crosses the sarcolemma and T-tubular membrane through the voltage gated L-type Ca^2+^ channels. This Ca^2+^ influx triggers the release of a larger quantity of Ca^2+^, called Ca^2+^ sparks, from the sarcoplasmic reticulum (SR), through the opening of SR Ca^2+^ release channels, known as ryanodine receptors (RyRs, particularly RyR2). This process is termed Ca^2+^-induced Ca^2+^ release. The combination of Ca^2+^ influx via the L-type Ca^2+^ channels and Ca^2+^ release from SR leads to the formation of cytosolic Ca^2+^ transients. The binding of cytosolic Ca^2+^ to the myofilaments then initiates cardiomyocyte contraction. Subsequent relaxation occurs by removal of Ca^2+^ from the cytosol mainly via the following mechanisms: (I) The SERCA2a re-uptakes the cytosolic Ca^2+^ back into the SR; the activity of this channel being modulated by its endogenous inhibitor phospholamban (PLN); (II) The Na^+^/Ca^2+^ exchanger (NCX) extrudes the Ca^2+^ out of the cells; (III) The mitochondrial Ca^2+^ uniporter transports Ca^2+^ into the mitochondria (4, 126).

Numerous studies have documented sex differences in cardiac EC coupling (127–129). For example, at rest, women have longer QT intervals and higher left ventricular ejection fraction than men (130–132). Other studies showed that ventricular myocytes in the female human failing heart have significantly greater contractility and enhanced L-type Ca^2+^ current (I_Ca, L_) compared to male patients (133–135). Studies in animal models also provide convincing evidence of sex differences in contractile function as observed in humans. It has been demonstrated that isolated cardiomyocytes from male rodents exhibit higher contraction than those from females (128, 136, 137). Furthermore, male rat cardiac myocyte and papillary muscle develop higher contractile force as well as significantly greater Ca^2+^ transient amplitude than females (138–142). In studies, using paced cardiomyocytes at the rates of 0.5–1.0 Hz, cardiac relaxation rate was slower in cardiomyocytes from female rats compared to aged matched males (139, 143).

The expression and function of cardiac L-type Ca^2+^ channels, which have a direct impact on the functional changes in EC coupling pathway in the heart, also show significant sexual dimorphisms. In adult cardiomyocytes, the Ca_v_α1C or Ca_v_1.2 (cardiac voltage-gated L-type Ca^2+^ channel) is the most abundant cardiac L-type Ca^2+^ channel which triggering cardiac contraction by regulation of I_Ca, L_ in cardiomyocytes (144–146). Therefore, it represents an important cellular site from which sex-based differences in myocardial intracellular Ca^2+^ handling and contractility may arise (138). Studies comparing the cardiac L-type Ca^2+^ Channel expression and I_Ca, L_ that have included both female and male animals, are still limited and the existing data are controversial. It has been demonstrated that the levels of L-type Ca^2+^ channel expression increase or do not change at all in the ventricle of female rats and rabbits in comparison to males (147–149). Similarly, comparative studies using isolated cardiomyocytes from female and male rats, mice, guinea pigs, and dogs showed that compared to males, the I_Ca, L_ density is either higher (147, 150–152) or lower in cells from females (153) or that there are no sex differences in I_Ca, L_ density at all (137, 140, 141, 154, 155). Even with these discrepancies in the data, which might be due to variations in the experimental protocols, species, and used strains, sex differences in the regulation and expression of L-type Ca^2+^ channels are apparent, although the underlying signaling mechanisms implicated in these sex differences are poorly understood.

In recent years, several studies provided evidence that the distal part of the C-terminus of the α1C subunit (α1C-dCT) of Ca_v_1.2 channel is proteolytically cleaved and shuttles between the plasma membrane and the nucleus of cardiomyocytes. It serves at the plasma membrane as an auto-inhibitor of Ca_v_1.2 channel activity (156–159), and acts as transcription factor in the nucleus, regulating the expression of different genes, including Ca_v_1.2 gene (CACNA1C) itself (160–163). Schroder et al. have provided evidence that the nuclear import of α1C-dCT in cardiomyocytes depresses Ca_v_1.2 transcription, while nuclear export of α1C-dCT increases Ca_v_1.2 channel activity consistent with a reduction of subsequent increase of Ca_v_1.2 gene transcription rates (161). In a recent study, we observed a remarkable sex-disparity in nuclear shuttling of α1C-dCT in mouse cardiomyocytes (164). Here, the nuclear shuttling was significantly higher in isolated female cardiomyocytes compared to males. Furthermore, we found a significant decrease in nuclear shuttling of α1C-dCT in both female and male cardiomyocytes upon serum withdrawal. However, subsequent E2-treatment normalized the intracellular distribution of α1C-dCT only in male cardiomyocytes. This effect of E2 was reversed by the ER-antagonist ICI 182,780, indicating the involvement of ER in this signaling pathway. These findings provide a possible explanation for the cellular mechanisms responsible for sex differences in the regulation of L-type Ca^2+^ channel in the heart, revealing the role of E2/ER in this process.

In addition to the L-type calcium channel, sexual dimorphisms in the expression, and activity of other cardiac calcium channels have also been reported. For example, several studies found that the expression and/or current of NCX (I_NCX_) are significantly higher in cardiomyocytes from female humans, rats, and rabbits compared to their male counterparts (135, 147–149, 165). Interestingly, Chen et al. showed that E2 administration increased NCX and I_NCX_ in female but not in male cardiomyocytes. These E2 effects appear to be mediated by a genomic mechanism involving the binding of E2 to its receptors, since these E2 effects were blunted by an ER antagonist (ICI 182,780) (165).

On the other hand, several studies have reported contradictory results on sex differences in the regulation of RyR2 expression and activity in the heart. It has been shown that the expression of RyR2 is higher in female rat cardiomyocytes compared to males (148, 149, 166), or that the expression does not differ in male and female rat and mice cardiomyocytes (155, 167). Bell et al. showed, however, that the regulation of RyR2 activity is different in male and female rat cardiomyocytes, with CaMKII (Ca^2+^/calmodulin-dependent protein kinase II)-mediated phosphorylation of RyR2 being lower in female cardiomyocytes than in male cardiomyocytes (167). This could be a possible explanation for the observed decrease in the gain of EC coupling (measured as SR Ca^2+^ release/Ca^2+^ current) in female rat and mice cardiomyocytes, which results from decreased size and duration of Ca^2+^ sparks by RyR2 (140, 155).

Collectively these findings suggest that the observed sex differences reflect, at least partly, the effects of E2 on myocardial Ca^2+^ handling, thus on contractility.

In this regard, studies with OVX rodents corroborate the effects of E2 on myocardial Ca^2+^ handling and contractility. Numerous studies with whole hearts or isolated cardiomyocytes from OVX mice, rats, rabbits, and pigs revealed that the E2 deficiency caused detrimental effects on both Ca^2+^ regulation and contractility of cardiomyocytes, such as enhanced Ca^2^+ transients, increased Ca^2^+ spark amplitudes, decreased myofilament Ca^2+^ sensitivity, and elevated contractions, in comparison to sham-operated controls (168–179). Remarkably, substitution of E2 effectively prevented the observed adverse effects (168, 169, 172, 174–179) and it could be shown that this is directly mediated via the ER by using the ER-antagonist ICI 182,780 (169).

In this context, several studies suggested that observed E2 effects are mediated by its receptors. Indeed, hearts of male ERKO-mice exhibit increased cardiac L-type Ca^2+^ channel expression and I_Ca, L_ (180), as well as significantly higher Ca^2+^ accumulation compared to control hearts during I/R (106). In line with these data, a recent study demonstrated that both E2 pre-treatment and/or ERα activation of Tet-on/ERα H9c2 cardiomyoblast cells inhibited isoproterenol-induced cytosolic Ca^2+^ accumulation in these cells, and this protective effect of the E2/ERα was reversed by treatment with a specific inhibitor of ERα (181). These data indicate that E2/ERα signaling pathway is involved in the regulation of Ca^2+^ balance in cardiomyocytes, thereby preventing the harmful effects of Ca^2+^ overload in the pathophysiology of the heart. By contrast, another study using ERKO- and BERKO-mice could not show that the inhibition of I_Ca, L_ and decrease in contraction depend on ERα or ERβ action (182). Moreover, it has been shown that in global GPER-KO mice, both left-ventricular contractility, and relaxation capacity were impaired only in males (122).

Furthermore, other studies have confirmed that the specific activation of different ER-isoforms affects cardiac contractility. Pelzer et al. showed that activation of ERα with the subtype-selective ERα agonist 16α-LE2 augments myocardial contractility to a measurable extent in OVX spontaneously hypertensive rats (183). Kulpa et al. showed that activation of ERα using the ERα agonist PPT (4,4',4”-(4-Propyl-[1H]-pyrazole-1,3,5-triyl) trisphenol) depressed actomyosin MgATPase activity and decreased myofilament Ca^2+^ sensitivity (184). Other studies have demonstrated the respective roles of ERβ and GPER activation in the regulation of SR Ca^2+^ handling proteins, such as SERCA2a and PLN, leading to improved contractility at the whole heart and single myocyte (66, 185).

These findings reveal that a solid understanding the roles of the various estrogen receptors in the regulation of cardiac contractility are needed in order to be able to find appropriate pharmacological agents that specifically target the receptors of interest.

## The Role of 17β-Estradiol and Estrogen Receptors in Cardiac Mitochondrial Function

Mitochondria are the main source of ATP and Reactive Oxygen Species (ROS) in the heart (186). It is considered that mitochondria play an essential role not only in regulation of cardiac contractility by providing ATP and by participating in Ca^2+^ homeostasis, but also they regulate cell death or apoptosis by ROS formation. Therefore, defects in mitochondrial structure and function are highly associated with CVDs (5, 186). E2 plays an important role in the supporting mitochondrial respiration, ATP production, and reducing ROS formation (Figure 1VI).

Sex differences in mitochondrial structure and function have been described. There is plenty of evidence that mitochondrial morphology and function differ between females and males in several organs and cell types. In the healthy mice hearts, although the female and male hearts displayed similar mitochondrial numbers, the proportion of large mitochondria (≥1 μm^2^) was significantly higher in female mice compared to males (56). Skeletal muscles from female rats show higher mitochondrial DNA and protein contents, as well as higher capacity of oxidative phosphorylation (OXPHOS) compared to male rats (187). Further, mitochondria in brain and liver from female mice exhibit higher antioxidant gene expression and lower oxidative damage under stress than in male animals (188). Additionally, several studies reported that the rate of ROS production is less in mitochondria from skeletal and cardiac muscle in female compared with aged matched male rats, particularly under stress conditions (187, 189, 190). Moreover, female rat hearts show altered posttranslational modification of several mitochondrial proteins under I/R in comparison to male hearts, including aldehyde dehydrogenase-2 (ALDH2) (189), a protein that has been reported to be involved in cardioprotective processes (191). Whole genome expression profiling performed in hearts of old (78-week) male and female Fischer 344 rats showed that a majority of genes involved in oxidative phosphorylation had higher expression in females compared to male rats (192). These studies suggest that E2 plays a role in the regulation of mitochondrial function, which is supported by evidence from several studies in OVX animals.

In particular, a high throughput quantitative proteomic approach with isolated mitochondria from left ventricles of OVX rat relative to ovary-intact hearts revealed that about 50% of the identified proteins altered in OVX rat cardiac mitochondria are involved in mitochondrial ATP production (193). Indeed, the observed reduction of protein subunits of the electron transport chain complex I (NADH dehydrogenase), II (succinate dehydrogenase), III (cytochrome bc1 complex), IV (cytochrome c oxidase), and V (F0F1 ATP-synthase) in E2-deficint hearts was associated with reduced ATP production that may contribute to increased I/R injury and disease risk with E2 deficiency in aged female rats. Interestingly, in a mouse model of a human hypertrophic cardiomyopathy (cTnT-Q92), E2-supplementation of OVX mice significantly elevated myocardial ATP levels and mitochondrial respiratory function compared to untreated OVX mice, thereby improving diastolic heart function (194). In another model of cardiomyopathy, hearts from OVX rats showed higher Ca^2+^ accumulation in their mitochondria, lower mitochondrial respiratory function, severely structurally damaged mitochondria, and increased myocardial cell death after I/R injury in comparison to intact animals (195). Again, in this study, E2-treatment of the hearts from OVX animals attenuated cardiac damage by I/R, and thereby maintained the LV function. Furthermore, mitochondria from hearts of OVX rats showed higher expression of apoptotic markers compared to mitochondria of intact animals (196). However, chronic E2-treatment of these animals significantly attenuated mitochondria-dependent apoptotic pathways. These data directly show that alterations in mitochondrial function are a highly selective myocardial response to E2 deficiency, and that E2-mediated cardioprotection at the level of the mitochondria leads to improved cardiac function.

Indeed, several studies demonstrated that E2 through its ERs affects the cardiac mitochondria directly via regulation of mitochondrial gene/protein expression. It has been shown that ERα and ERβ are localized in the mitochondria of cardiac cells (62, 197–199). The presence of ERs in the mitochondria of cardiac cells suggests that they mediate the observed protective effects of E2, at least partly, by regulating mitochondrial structure and function in the heart. In line with the role of ERα and ERβ as transcription factors, distinct evidence supports the notion that mitochondrial DNA (mtDNA) could be one of the major targets for E2 acting via ER in cardiac cells. This is supported, for example, (1) by the presence of putative ERE on the mtDNA (200–202), (2) the E2-induced up-regulation of several mitochondrial-encoded genes, such as COXI and COXII (cytochrome c oxidase subunits I and II) (203, 204), and (3) the E2-induced expression of several nuclear-encoded mitochondrial genes, such as NRF-1 (nuclear respiratory factor 1), NRF-2 (nuclear respiratory factor 2), TFAM (mitochondrial transcription factor), PGC-1α (peroxisome proliferator-activated receptor gamma co-activator-1 alpha), and MEF2a (Myocyte enhancer factor 2A) (56, 202, 205, 206), whose proteins translocate into the mitochondria and thereby influence mitochondrial function. Additionally, it could be shown that in rat myocardium after severe hemorrhage the E2-induced increased expression of these genes was associated with an increase in COX IV (cytochrome c oxidase subunit IV), mtDNA-encoded COX I (cytochrome c oxidase subunit I), ATP synthase β-subunit, and mitochondrial ATP (207, 208). All these effects were abolished with the ER antagonist ICI 182,780, indicating an ER-specific effect.

The role of E2 and ER in the regulation of mitochondrial structure and function is established from studies with ER deficient mouse models. Microarray analysis using ERKO- and BERKO-mice showed that E2/ERβ pathways mediate down-regulation of mRNAs for nuclear-encoded subunits in each of the major complexes of the electron transport chain, whereas ERα is essential for most of the E2-mediated increase in gene expression including electron transport chain proteins and proteins involved in the anti-oxidative stress response (209). In a mouse model of exercise-induced physiological myocardial hypotrophy, we demonstrated that only female WT-mice showed an increase in the expression of key regulators of mitochondrial function e.g., NRF-1,−2, Mef2a, Atp5k (subunit E of mitochondrial F1F0-ATP synthase), and electron transport chain proteins (complexes I, III, and V) after running. Interestingly, ERβ deletion abolished the observed effects (56). Additionally, our study also showed that the activated ERβ significantly increased the expression of MEF2A, NRF-1, and−2 genes in a cardiomyocyte cell line (AC16 cells) (56). In line with these data, the expression of NRF-1 is diminished in BERKO hearts (209). On the other hand, Zhai et al. demonstrated that ERKO-mice hearts showed marked mitochondrial damages (fragmented and swollen mitochondria) and severe impairment of mitochondrial respiratory function compared to control hearts after I/R (106). To our knowledge a direct localization of GPER within the mitochondria has not been documented so far. However, analysis of DNA microarray data followed by Gene Set Enrichment Analysis (GSEA) from female and male cardiomyocytes of WT- and csGPER-KO-mice revealed that mitochondrial genes are enriched only in csGPER-KO females (124, 125), which provided direct evidence that the cardioprotective effects of GPER under physiological and pathological conditions in the female csGPER-KO-mice may be related to enhancements in mitochondrial function.

Several studies demonstrated that E2 also indirectly affects the cardiac mitochondria via regulation of ROS production. Elevated Ca^2+^ uptake by mitochondria results in the opening of the mitochondrial permeability transition pore (mPTP) and enhanced release of cytochrome c accompanied by dramatic increase in ROS formation, which leads to cell death via the induction of apoptosis pathways (210, 211). It has been shown that in comparison to male, mitochondria from female rat hearts accumulate Ca^2+^ more slowly (212), which might represent a mechanism that may underlie, at least partly, sex-related differences accounting for females to suffer less injury with I/R. Indeed, several studies demonstrated that E2 administration can acutely attenuate the Ca^2+^ accumulation in mitochondria, inhibit Ca^2+^-induced opening of mPTP in isolated heart mitochondria, prevent Ca^2+^-induced release of cytochrome c from mitochondria, and inhibit ischemia-induced apoptosis in perfused heart (213–215). Interestingly, Feng et al. demonstrated that post-ischemic E2 administration to both male and OVX-female rats preserved mitochondrial structural integrity, which was associated with an increased tolerance to Ca^2+^ overload or augmented mitochondrial Ca^2+^ retention capacity (216) which reflects an inhibition of the mPTP opening in both male and OVX-female animals.

Here again, using ER deficient mice could be shown that these E2 effects are mediated by ERs. Male ERKO hearts subjected to I/R showed an accumulated Ca^2+^ deposition in their mitochondria which led to severe mitochondrial damage (fragmented and swollen mitochondria) in cardiomyocytes, and consequently to the depletion of ATP production (106). Using ERKO-, BERKO-, and ERα and ERβ double knockout (DERKO)-mice, Luo et al. found that both ER subtypes are necessary for E2-mediated cardioprotection during I/R in female hearts. Thereby, E2 and ER upregulate mitochondrial p38β-MAPK activity, with subsequent phosphorylation of the MnSOD (manganese superoxide dismutase), leading to enhanced SOD activity, thereby minimizing mitochondrial-derived ROS production and reduction of myocardial infarct size post I/R (217). By contrast, a systematic analysis of WT-, ERKO-, BERKO-, and GPER-KO-mice subjected to I/R showed that only GPER expression is essential for the acute action of E2 in cardioprotection against I/R injury in male mouse via a cascade involving PKC translocation, ERK1/2/GSK-3β (Glykogensynthase-Kinase 3β)- phosphorylation leading to the inhibition of the mPTP opening, resulting in reduction of harmful mitochondrial ROS generation (80). However, a pre-administration with G15, a specific GPER antagonist, reversed this estrogenic effect. This data indicate that GPER activation mediates E2-induced increase in mitochondrial Ca^2+^ retention capacity, and the GPER-mediated cardioprotective effect of post-ischaemic E2 is related to a decrease in mPTP sensitivity to Ca^2+^ overload, a process which is mediated via activation of the MEK/ERK/GSK-3β axis.

These data suggest that depending on the time period of E2-treatment, sex, and species different ERs can be activated by E2, which mediate the mitochondrial-dependent cardioprotective effect of E2 against I/R injury.

## Conclusion

In the past, most clinical and animal studies did not include both sexes or differentiate between sexes in the data analysis. This might be the possible reason that our understanding of the molecular and cell-based mechanisms underlying sex-based differences in cardiovascular system are still incomplete so far. A more thorough understanding of underlying sex-dimorphic mechanisms in cardiac health and disease is required to effectively treat patients with CVDs. The presented data in this review support the concept that sex specific regulation of cardiac Ca^2+^ ion channels and mitochondrial function by E2 and ERs could be, at least partly, responsible for differences in cardiovascular disease incidence and outcomes. However, further attempts toward a more detailed understanding of E2 and ERs roles in the heart are needed to develop new drugs that target the beneficial effects on CVD in both sexes.

## Author Contributions

All authors listed have made a substantial, direct and intellectual contribution to the work, and approved it for publication.

### Conflict of Interest Statement

The authors declare that the research was conducted in the absence of any commercial or financial relationships that could be construed as a potential conflict of interest.
